# Effects of Intravitreal Anti-VEGF Therapy on Conjunctival Flora and Antibiotic Resistance in Age-Related Macular Degeneration

**DOI:** 10.3390/microorganisms14081735

**Published:** 2026-08-07

**Authors:** Ayten Gündüz, Zarife Ekici Gök, Özge Evren

**Affiliations:** 1 Department of Medical Microbiology, Faculty of Medicine, Malatya Turgut Özal University, Malatya 44210, Türkiye; aytengunduz@hotmail.com; 2 Department of Ophthalmology, Faculty of Medicine, Malatya Turgut Özal University, Malatya 44210, Türkiye; 3 Department of Ophthalmology, Malatya Training and Research Hospital, Malatya 44330, Türkiye; drozgeevren@hotmail.com

**Keywords:** age-related macular degeneration, anti-VEGF therapy, intravitreal injection, conjunctival microbiota, antibiotic resistance

## Abstract

Background: Age-related macular degeneration (AMD) is a leading cause of visual impairment among older adults. Intravitreal anti-vascular endothelial growth factor (anti-VEGF) agents are widely used for neovascular AMD; however, their potential effects on the conjunctival microbiota remain poorly understood. This study evaluated the impact of intravitreal anti-VEGF therapy on conjunctival microbial composition and antimicrobial resistance patterns. Materials and Methods: Ninety patients with AMD were enrolled and assigned to three groups: bevacizumab-treated (*n* = 30), aflibercept-treated (*n* = 30), and untreated dry-type AMD controls (*n* = 30). Conjunctival swab samples were collected two months after the last injection in treated patients and during routine examination in controls. Bacterial identification and antimicrobial susceptibility testing were performed using automated microbiological systems. Results: Culture positivity rates were comparable among groups, although a lower growth rate was observed in the bevacizumab group (83.3%) than in the aflibercept and control groups (100% each). *Staphylococcus epidermidis* was significantly more prevalent in both treatment groups than in controls (*p* = 0.014). Most other bacterial species were detected more frequently in controls. Levofloxacin resistance among staphylococcal isolates was significantly higher in the aflibercept group (*p* = 0.007), whereas erythromycin and fusidic acid resistance were significantly higher in controls (*p* = 0.040 and *p* = 0.001, respectively). Resistance in treated eyes was predominantly associated with *S. epidermidis* and *S. hominis*. Conclusions: Intravitreal anti-VEGF therapy may be associated with transient alterations in conjunctival microbiota composition and antimicrobial resistance patterns. These findings may have implications for infection prevention in patients undergoing repeated intravitreal injections.

## 1. Introduction

Age-related macular degeneration (AMD) is an important retinal disorder that typically occurs in older adults and represents one of the leading causes of irreversible visual impairment after cataract and glaucoma. The disease results in central vision loss due to degeneration of the choriocapillaris within the macular region [1,2]. AMD has two major clinical forms: neovascular (exudative) and dry (atrophic, non-exudative). The neovascular form accounts for approximately 10% of cases and is characterized by abnormal macular neovascularization, whereas the dry form constitutes nearly 90% of patients and is associated with photoreceptor damage and slowly progressive visual decline [3].

Vascular endothelial growth factor (VEGF), a key mediator of pathological neovascularization, plays a central role in AMD pathogenesis [4,5]. Increased ocular VEGF levels have been demonstrated in patients with AMD [4], leading to the widespread use of anti-VEGF agents in the management of neovascular AMD. Intravitreal anti-VEGF injections inhibit abnormal retinal vascular proliferation and help slow the progression of vision loss [6,7].

Bevacizumab and aflibercept are among the most commonly used anti-VEGF agents for neovascular AMD [8]. Although administered intravitreally at low doses, these agents may enter the systemic circulation to a limited extent, potentially altering systemic VEGF levels and causing undesirable systemic effects, particularly involving cardiovascular and cerebrovascular systems [9,10]. In addition, the vascular and immunomodulatory effects of anti-VEGF therapy may induce biological changes in the skin, mucosal tissues, and other organs [11,12].

In this study, we investigated the effects of intravitreal bevacizumab and aflibercept on conjunctival flora, considering their potential systemic and local (direct and indirect) biological effects. Data directly comparing these agents with an untreated dry-type AMD control group using a delayed sampling strategy are limited, and our findings add to the emerging literature on ocular surface microbiota following repeated intravitreal anti-VEGF therapy.

## 2. Materials and Methods

### 2.1. Study Settings

This study was conducted at the Departments of Ophthalmology and Medical Microbiology, Malatya Turgut Ozal University Faculty of Medicine Training and Research Hospital. The study protocol was approved by the Malatya Turgut Ozal University Health Sciences Scientific Research Ethics Committee (Decision No. 2025/121; Date: 14 May 2025). All study procedures were conducted in accordance with the principles of the Declaration of Helsinki.

### 2.2. Study Population and Groups

Patients diagnosed with age-related macular degeneration (AMD) and followed in the retina clinic were enrolled. The study consisted of three groups of 30 patients each (total *n* = 90):

(1) Patients receiving intravitreal bevacizumab (Altuzan^®^);
(2) Patients receiving intravitreal aflibercept (Eylea^®^);
(3) Untreated dry-type AMD patients serving as the control group.

To minimize conjunctival microbiota variability related to different etiologies, the control group was selected from AMD patients who had not received intravitreal therapy, ensuring a comparable disease background across groups. All participants were informed about the study, and written informed consent was obtained prior to inclusion.

### 2.3. Inclusion Criteria

Patients were included if they met the following criteria:

- A confirmed diagnosis of AMD;
- Aged ≥ 50 years;
- Had not used topical or systemic antibiotics within the previous 2 months;
- No signs of active ocular infection or inflammation;
- Completion of at least six intravitreal anti-VEGF injections before conjunctival sampling;
- Had not undergone ocular surgery within the previous 2 months.

### 2.4. Exclusion Criteria

Patients were excluded if they met the following criteria:

- Systemic antibiotic use within the last 2 months;
- Diabetes mellitus, rheumatologic disease, or immunodeficiency;
- Contact lens use;
- Eyelid abnormalities;
- Ocular surface disease such as clinically significant dry eye (Schirmer I <10 mm) or blepharitis;
- Ocular surgery within the previous 2 months.

### 2.5. Intravitreal Injection Protocol

Intravitreal injections were administered through the pars plana using the standard three-dose loading regimen at 1-month intervals. The intravitreal doses were 1.25 mg/0.05 mL for bevacizumab and 2 mg/0.05 mL for aflibercept.

All injections were performed in an operating room under sterile conditions following topical anesthesia. Periocular skin and the conjunctival surface were disinfected with 5% povidone–iodine and irrigated with sterile saline. Each eye received 0.05 mL of the assigned anti-VEGF agent through the pars plana using a 30-gauge needle, and the injection was administered slowly over approximately 3–5 s.

Following the procedure, all patients received the same post-injection topical broad-spectrum antibiotic prophylaxis for 1 week (ofloxacin 0.3% four times daily) and were monitored according to routine clinical follow-up protocols.

### 2.6. Sample Collection

The two-month sampling interval was chosen to allow the transient effects of peri-injection povidone–iodine and short-term topical antibiotic prophylaxis to subside, thereby better reflecting the more persistent local and systemic biological impact of anti-VEGF therapy on conjunctival microbiota. In the control group, conjunctival samples were collected from a randomly selected eye during routine clinical examination. In treated patients, samples were obtained from the injected eye.

All participants underwent comprehensive ophthalmic examination by an ophthalmologist. To avoid potential inhibitory effects on microorganisms, topical anesthetic agents were not administered prior to sampling. The lower eyelid was gently retracted, and a sterile cotton swab was used to obtain a conjunctival specimen from the inferior fornix while carefully avoiding contact with eyelashes or eyelid margins. The swabs were immediately placed into Stuart transport medium (CITOSWAB, Jiangsu, China) and promptly delivered to the microbiology laboratory for analysis.

### 2.7. Microbiological Analysis

Upon arrival at the microbiology laboratory, conjunctival swab samples were promptly inoculated into thioglycolate broth (RTA, Kocaeli, Türkiye) for enrichment and recovery of organisms present at low inoculum and incubated at 37 °C for 24 h. Subsequently, under biosafety level-2 cabinet conditions, subcultures were performed on 5% sheep blood agar, chocolate agar, eosin methylene blue (EMB) agar, and Sabouraud dextrose agar (SDA) media (RTA, Kocaeli, Türkiye).

Culture plates were incubated at 37 °C for 24 h, and plates without visible growth were further incubated for up to 48 h. For fungal detection, SDA plates were incubated for up to 3 weeks. Colonies growing on culture media were subcultured to obtain pure isolates. Preliminary identification was performed using Gram staining and conventional biochemical tests, including catalase, coagulase, pyrrolidonyl arylamidase (PYR), and oxidase tests. Final species-level identification of bacterial and yeast isolates was achieved using a fully automated identification system (VITEK-2 Compact^®^, bioMérieux, Marcy-l’Étoile, France).

### 2.8. Antimicrobial Susceptibility Testing

Antimicrobial susceptibility testing of *Staphylococcus* isolates identified at the species level was performed using the automated VITEK^®^ 2 Compact system, and minimum inhibitory concentration (MIC) values were determined. Methicillin resistance was assessed based on the MIC value for cefoxitin. Because *Staphylococcus* species represented the predominant bacterial isolates in our study and are the most common causative organisms of post-injection ocular infections, antimicrobial susceptibility testing was performed only for *Staphylococcus* isolates. Among fluoroquinolones, the VITEK^®^ 2 Compact antimicrobial susceptibility panel for *Staphylococcus* isolates reports only levofloxacin. Therefore, levofloxacin susceptibility was evaluated as the representative fluoroquinolone in the present study. Susceptibility results were interpreted according to the criteria of the European Committee on Antimicrobial Susceptibility Testing (EUCAST) [13].

### 2.9. Statistical Analysis

Descriptive statistics were presented as mean ± standard deviation for continuous variables and as number (*n*) and percentage (%) for categorical variables.

Normality was assessed using the Shapiro–Wilk test and visual inspection of histograms and Q–Q plots. Comparisons of continuous variables among the three groups were performed using one-way analysis of variance (ANOVA) for normally distributed data or the Kruskal–Wallis test for non-normally distributed data.

Associations between categorical variables were analyzed using the chi-square test or Fisher’s exact test, as appropriate.

A two-sided *p* value < 0.05 was considered statistically significant. All statistical analyses were performed using SPSS statistical software (IBM SPSS for Windows, version 27.0; IBM Corp., Armonk, NY, USA).

### 2.10. Power Analysis

The sample size of this study was determined by an a priori power analysis. According to calculations performed using G*Power software (version 3.1), assuming an effect size (Cohen’s w) of 0.53, a significance level (α) of 0.05, and a statistical power (1 − β) of 0.95, the required total sample size was estimated to be 60 participants (*n* = 20 per group). Considering potential dropouts and culture-negative samples, 30 patients were included in each group, increasing the total sample size to 90.

## 3. Results

When the demographic characteristics of the study groups were evaluated, no statistically significant differences were observed among the groups in terms of mean age or sex distribution (*p* > 0.05). Similarly, conjunctival culture positivity rates did not differ significantly between the groups; however, the culture growth rate was lower in the bevacizumab group compared with the aflibercept and control groups (Table 1).

**Figure 1:**
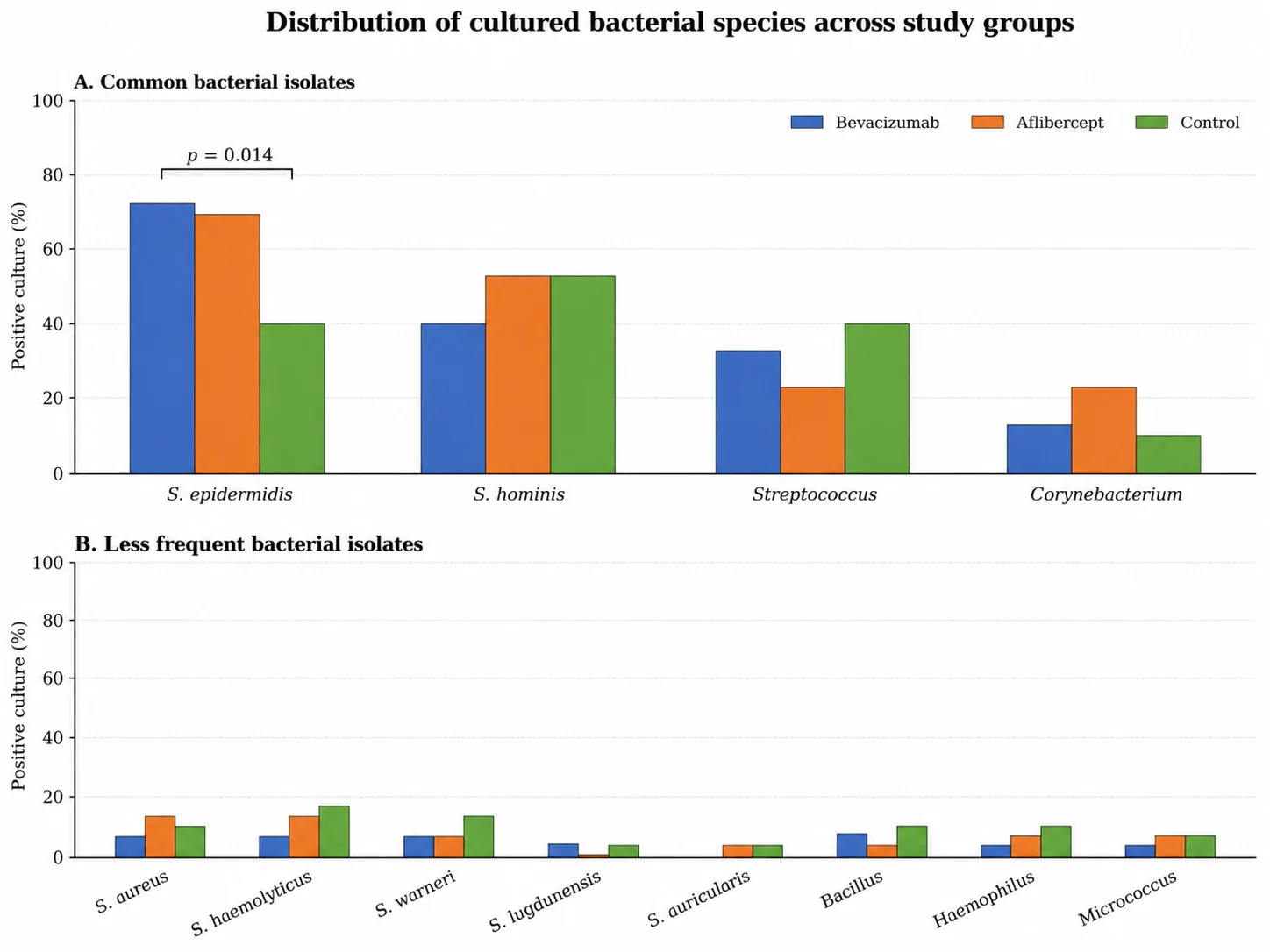
Distribution of cultured bacterial species across the study groups. (**A**) Common bacterial isolates. (**B**) Less frequent bacterial isolates. Values represent the percentage of positive cultures. *Staphylococcus epidermidis* was the only species that differed significantly among the groups (*p* = 0.014).

Comparison of the three groups with respect to isolated bacterial species demonstrated a statistically significant difference only for *Staphylococcus epidermidis* (*p* = 0.014). The prevalence of *S. epidermidis* was significantly higher in both the bevacizumab and aflibercept groups compared with the control group. No statistically significant differences were detected among the groups for any other bacterial species (all *p* > 0.05) (Table 2, Figure 1).

**Figure 2:**
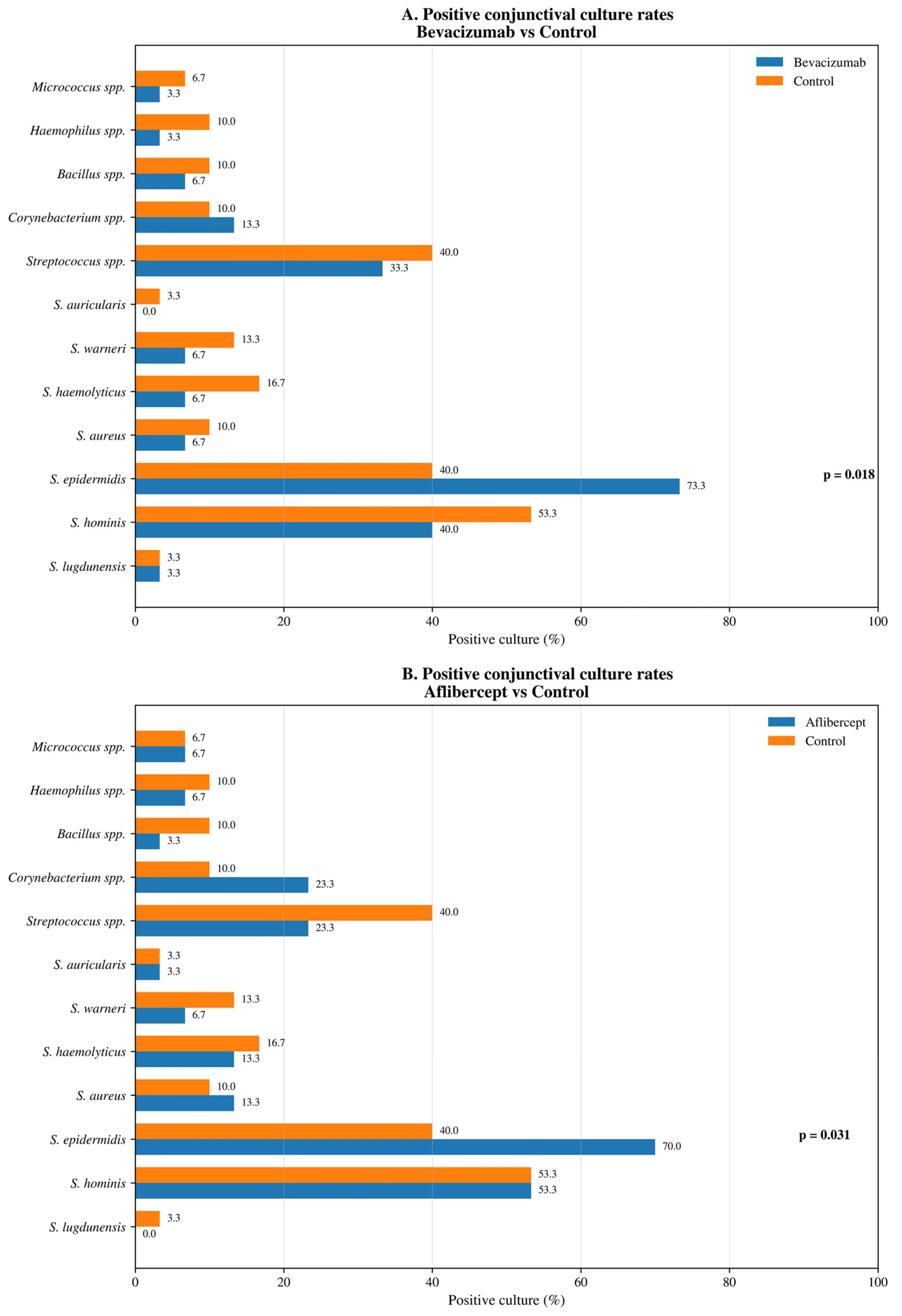
Comparison of positive conjunctival bacterial culture rates between the treatment and control groups. (**A**) Bevacizumab versus control. (**B**) Aflibercept versus control. Data are expressed as the percentage of eyes with positive cultures for each bacterial species. Statistically significant differences were observed only for *Staphylococcus epidermidis* (*p* = 0.018 for bevacizumab vs. control; *p* = 0.031 for aflibercept vs. control).

When the bevacizumab and aflibercept groups were each compared separately with the control group, *Staphylococcus epidermidis* remained significantly more frequent in both treatment groups (*p* = 0.014 and *p* = 0.031, respectively). No significant differences were found for the remaining bacterial species (all *p* > 0.05). Although not statistically significant, the overall trend indicated higher bacterial growth rates in the control group for most organisms except *Corynebacterium* spp. Conversely, *Corynebacterium* spp. and *Staphylococcus aureus* were relatively more frequent in the treatment groups, although these differences did not reach statistical significance (Table 3, Figure 2A,B).

Comparison of antibiotic resistance profiles among staphylococcal isolates revealed that levofloxacin resistance was significantly higher in the aflibercept group (*p* = 0.007). Although levofloxacin resistance was also numerically higher in the bevacizumab group than in controls, this difference was not statistically significant. In contrast, resistance to erythromycin and fusidic acid was significantly higher in the control group (*p* = 0.040 and *p* = 0.001, respectively). No vancomycin resistance was detected in any group. Methicillin resistance did not differ significantly among the groups (*p* > 0.05) (Table 4).

At the species level, analysis within the bevacizumab group showed that only levofloxacin resistance varied significantly by bacterial species (*p* = 0.045), with resistance predominantly concentrated in *Staphylococcus hominis* and *Staphylococcus epidermidis*. Although methicillin resistance did not show statistical significance, it was also more frequently observed in these two species. Resistance to other antibiotics similarly tended to cluster in *S. hominis* and *S. epidermidis*, whereas no resistance to linezolid, vancomycin, or tigecycline was detected.

In the aflibercept group, erythromycin resistance was mainly concentrated in *Staphylococcus hominis* and *Staphylococcus epidermidis* and was significantly higher, particularly for *S. hominis* (*p* = 0.014). Tetracycline resistance also showed a significant increase and was similarly clustered in *S. epidermidis* and *S. hominis*.

In the control group, no statistically significant association was found between bacterial species and antibiotic resistance for any tested antibiotic (all *p* > 0.05). Resistance distribution appeared relatively homogeneous across species. Although resistance was most frequently observed in *Staphylococcus hominis* and *Staphylococcus epidermidis*, this predominance did not constitute a statistically significant clustering. Methicillin resistance likewise showed no species-specific significant difference but was again more commonly detected in these two species.

Overall, antibiotic resistance in the intravitreal anti-VEGF treatment groups appeared to concentrate predominantly in coagulase-negative staphylococci, particularly *Staphylococcus hominis* and *Staphylococcus epidermidis*, whereas the control group demonstrated a more balanced resistance distribution. These findings suggest that repeated intravitreal interventions may exert selective pressure on the ocular surface microbiota, potentially facilitating resistance development in specific staphylococcal species.

## 4. Discussion

In patients with neovascular age-related macular degeneration, anti-vascular endothelial growth factor agents administered as intravitreal injections, such as bevacizumab and aflibercept, inhibit VEGF-mediated endothelial cell proliferation and neovascularization [14,15]. Systemic and local ophthalmic adverse effects of intravitreal anti-VEGF agents have been reported. Systemic effects may develop due to the limited penetration of these agents into the systemic circulation [16,17]. The accumulation of bevacizumab and aflibercept in the body may persist over time, and measurable serum drug levels have been detected for at least one month after intravitreal injection. The systemic effects of bevacizumab have been reported to be greater than those of aflibercept [17,18].

The conjunctival microbiota may vary depending on both intrinsic and extrinsic factors [19]. Intravitreal agents are known to exert effects on vascular structures and the immune system [20,21,22]. In this study, we examined the potential changes in the conjunctival microbiota that may occur due to the systemic and local effects of intravitreal drugs on conjunctival vascular circulation.

Previous microbiota studies in patients receiving intravitreal injections have reported variable culture positivity rates. Hsu et al. reported a 77% culture positivity rate in conjunctival samples obtained from eyes receiving intravitreal injections [23], whereas another study found negative cultures in 16 of 148 samples (10.81%) [24]. Yin et al. demonstrated higher culture positivity in the patient group (38.2%) compared with controls (19.4%) [25]. Kaldırım et al. reported growth rates of 40.5%, 50%, and 65.5% across three groups [26]. In the present study, although no statistically significant difference in culture growth rates was observed among groups, the bevacizumab group showed a lower growth rate compared with the other groups (83.3%, 100%, and 100%, respectively). This finding may be related to the more pronounced systemic biological effect of bevacizumab compared with aflibercept, potentially resulting in suppression of the conjunctival microbiota.

Coagulase-negative staphylococci are the most common components of the normal conjunctival microbiota [27]. Studies evaluating conjunctival cultures after intravitreal injection have likewise identified coagulase-negative staphylococci as the predominant flora [24,28,29]. Consistent with the literature, coagulase-negative staphylococci were the most frequently isolated bacterial group in both treatment and control groups in the present study.

Previous studies have reported *Staphylococcus epidermidis* as the most frequently isolated coagulase-negative staphylococcal species following intravitreal injection [24,30]. In our study, *S. epidermidis* was significantly more prevalent in both bevacizumab- and aflibercept-treated groups compared with the control group. Moreover, apart from *S. epidermidis* and *Corynebacterium* spp., other bacterial species tended to be more frequently detected in the control group, although without statistical significance. This observation may be explained by reduced conjunctival vascular perfusion and local oxygenation associated with anti-VEGF therapy, potentially altering microbial survival conditions. Consequently, anti-VEGF treatment may lead to an overall reduction in microbial diversity and species richness.

Regarding the distribution of staphylococcal species, Budzinskaya et al. identified *S. epidermidis* as the most common species (66.1%), followed by *S. aureus*, *S. hominis*, and *S. haemolyticus* [30]. Another study also reported *S. aureus* as the second most frequent species after *S. epidermidis* [26]. Similarly, *S. epidermidis* was the most frequently isolated species in all three groups in our study, followed by *S. hominis*.

Studies evaluating other conjunctival bacterial species have generally shown that *S. aureus* and *Corynebacterium* spp. are the most common organisms after coagulase-negative staphylococci [24,25,28,29]. Another report identified *Corynebacterium* spp., *Streptococcus* spp., and *S. aureus* as the most frequent non-staphylococcal organisms [26]. In the present study, *Streptococcus* spp. were the most frequent non-staphylococcal isolates in treatment groups, followed by *Corynebacterium* spp., while *Streptococcus* spp. were likewise the most common in the control group.

Increased resistance to fluoroquinolones and macrolides has been reported in conjunctival microbiota studies [31,32,33]. Kim et al. demonstrated significantly higher fluoroquinolone and macrolide resistance in eyes receiving intravitreal injections compared with controls [34]. De Miranda et al. also reported elevated fluoroquinolone resistance among staphylococci [24], whereas Hsu et al. did not detect resistance to macrolides or fluoroquinolones [23]. In our study, levofloxacin resistance was significantly higher in the aflibercept group compared with both bevacizumab and control groups (*p* = 0.007). Although not statistically significant, levofloxacin resistance was also higher in the bevacizumab group than in controls. Within-group analyses showed that, in the bevacizumab group, levofloxacin resistance differed significantly by bacterial species (*p* = 0.045) and was concentrated in *S. epidermidis* and *S. hominis*. A similar clustering pattern was observed in the aflibercept and control groups despite the absence of statistical significance. This pattern suggests that repeated anti-VEGF injections may exert selective pressure favoring fluoroquinolone resistance on the ocular surface.

Conversely, macrolide resistance was significantly higher in the untreated control group (*p* = 0.040). In the control group, antibiotic resistance distribution among staphylococcal species was homogeneous, and no statistically significant association was observed between species and resistance for any tested antibiotic (all *p* > 0.05).

### Limitations

The present study has several limitations, including its single-center design and relatively small sample size. Additionally, the evaluation of only aerobic bacteria in the conjunctival microbiota represents another limitation. Larger, multicenter studies are needed to clarify the long-term effects of anti-VEGF therapy on the conjunctival microbiota.

The absence of baseline conjunctival cultures before anti-VEGF treatment limits our ability to determine whether the observed microbial changes were pre-existing or treatment-related.

In addition, the exact cumulative number of previous intravitreal injections was not available. Therefore, the potential influence of differences in cumulative povidone-iodine and antibiotic exposure between treatment groups could not be evaluated.

Another limitation of this study is that the control group consisted of patients with dry AMD rather than untreated neovascular AMD. Although untreated neovascular AMD patients would provide a more comparable disease control group, withholding anti-VEGF treatment solely for study purposes would not be ethically appropriate. Therefore, the observed differences may partly reflect differences between AMD subtypes in addition to the potential effects of repeated intravitreal anti-VEGF therapy.

Since conjunctival cultures were assessed at only one post-treatment time point, the present findings primarily reflect short-term changes after intravitreal anti-VEGF therapy. Whether these microbiological alterations persist or return to baseline over time remains uncertain and should be investigated in future longitudinal studies.

## 5. Conclusions

In conclusion, we observed limited but statistically significant differences in selected bacterial species and resistance profiles among groups. These findings may reflect, at least in part, the vascular and immunomodulatory effects of intravitreal anti-VEGF therapy on the ocular surface. Awareness of potential microbiota shifts and resistance patterns could help inform empirical management strategies for infections in patients undergoing repeated intravitreal injections.

## Figures and Tables

**Table 1:** Comparison of demographic characteristics and culture positivity among groups.

Variable	Bevacizumab Group (*n* = 30)	Aflibercept Group (*n* = 30)	Control Group (*n* = 30)	*p* Value
Age (years), mean ± SD	74.7 ± 9.7	73.4 ± 7.4	70.5 ± 7.3	0.135 ^†^
Sex, *n* (%)				0.553 ^‡^
Male	11 (36.7%)	12 (40.0%)	15 (50.0%)	
Female	19 (63.3%)	18 (60.0%)	15 (50.0%)	
Culture growth, *n* (%)				0.064 ^‡^
Present	25 (83.3%)	30 (100%)	30 (100%)	
Absent	5 (16.7%)	0 (0.0%)	0 (0.0%)	

Notes ^†^ One-way ANOVA or Kruskal–Wallis test, as appropriate. ^‡^ Chi-square test. SD: standard deviation.

**Table 2:** Distribution of cultured bacterial species across study groups.

Bacterial Species	Bevacizumab Group *n* (%)	Aflibercept Group *n* (%)	Control Group *n* (%)	*p* Value
*Staphylococcus lugdunensis*	1 (3.3%)	0 (0.0%)	1 (3.3%)	0.600
*Staphylococcus hominis*	12 (40.0%)	16 (53.3%)	16 (53.3%)	0.491
*Staphylococcus epidermidis*	22 (73.3%)	21 (70.0%)	12 (40.0%)	**0.014**
*Staphylococcus aureus*	2 (6.7%)	4 (13.3%)	3 (10.0%)	0.690
*Staphylococcus haemolyticus*	2 (6.7%)	4 (13.3%)	5 (16.7%)	0.484
*Staphylococcus warneri*	2 (6.7%)	2 (6.7%)	4 (13.3%)	0.578
*Staphylococcus auricularis*	0 (0.0%)	1 (3.3%)	1 (3.3%)	0.600
*Streptococcus* spp.	10 (33.3%)	7 (23.3%)	12 (40.0%)	0.380
*Corynebacterium* spp.	4 (13.3%)	7 (23.3%)	3 (10.0%)	0.333
*Bacillus* spp.	2 (6.7%)	1 (3.3%)	3 (10.0%)	0.585
*Haemophilus* spp.	1 (3.3%)	2 (6.7%)	3 (10.0%)	0.585
*Micrococcus* spp.	1 (3.3%)	2 (6.7%)	2 (6.7%)	0.809

Note: *p* values were calculated using the chi-square or Fisher’s exact test, as appropriate. Bold indicates statistical significance (*p* < 0.05).

**Table 3:** Comparison of cultured bacterial growth between treatment groups and the control group.

Bacterial Species	Bevacizumab *n* (%)	Control *n* (%)	*p* Value	Aflibercept *n* (%)	Control *n* (%)	*p* Value
*Staphylococcus lugdunensis*	1 (3.3%)	1 (3.3%)	1.000	0 (0.0%)	1 (3.3%)	1.000
*Staphylococcus hominis*	12 (40.0%)	16 (53.3%)	0.366	16 (53.3%)	16 (53.3%)	1.000
*Staphylococcus epidermidis*	22 (73.3%)	12 (40.0%)	0.018	21 (70.0%)	12 (40.0%)	0.031
*Staphylococcus aureus*	2 (6.7%)	3 (10.0%)	1.000	4 (13.3%)	3 (10.0%)	1.000
*Staphylococcus haemolyticus*	2 (6.7%)	5 (16.7%)	0.453	4 (13.3%)	5 (16.7%)	1.000
*Staphylococcus warneri*	2 (6.7%)	4 (13.3%)	0.683	2 (6.7%)	4 (13.3%)	0.683
*Staphylococcus auricularis*	0 (0.0%)	1 (3.3%)	1.000	1 (3.3%)	1 (3.3%)	1.000
*Streptococcus* spp.	10 (33.3%)	12 (40.0%)	0.670	7 (23.3%)	12 (40.0%)	0.356
*Corynebacterium* spp.	4 (13.3%)	3 (10.0%)	1.000	7 (23.3%)	3 (10.0%)	0.344
*Bacillus* spp.	2 (6.7%)	3 (10.0%)	1.000	1 (3.3%)	3 (10.0%)	0.625
*Haemophilus* spp.	1 (3.3%)	3 (10.0%)	0.625	2 (6.7%)	3 (10.0%)	1.000
*Micrococcus* spp.	1 (3.3%)	2 (6.7%)	1.000	2 (6.7%)	2 (6.7%)	1.000

**Table 4:** Distribution of antibiotic resistance among *Staphylococcus* species across study groups.

Antibiotic	Susceptibility	Bevacizumab *n* (%)	Aflibercept *n* (%)	Control *n* (%)	*p* Value
FOX (Cefoxitin)	Susceptible	22 (48.9%)	26 (48.1%)	20 (41.7%)	0.737
	Resistant	23 (51.1%)	28 (51.9%)	28 (58.3%)	
OKS (Oxacillin)	Susceptible	22 (48.9%)	26 (48.1%)	20 (41.7%)	0.737
	Resistant	23 (51.1%)	28 (51.9%)	28 (58.3%)	
LEV (Levofloxacin)	Susceptible	25 (55.6%)	24 (44.4%)	36 (75.0%)	**0.007**
	Resistant	20 (44.4%)	30 (55.6%)	12 (25.0%)	
E (Erythromycin)	Susceptible	26 (57.8%)	33 (61.1%)	18 (37.5%)	**0.040**
	Resistant	19 (42.2%)	21 (38.9%)	30 (62.5%)	
DA (Clindamycin)	Susceptible	40 (88.9%)	43 (79.6%)	39 (81.3%)	0.439
	Resistant	5 (11.1%)	11 (20.4%)	9 (18.7%)	
LNZ (Linezolid)	Susceptible	45 (100%)	52 (96.3%)	45 (93.8%)	0.248
	Resistant	0 (0.0%)	2 (3.7%)	3 (6.2%)	
DAP (Daptomycin)	Susceptible	42 (93.3%)	51 (94.4%)	43 (89.6%)	0.628
	Resistant	3 (6.7%)	3 (5.6%)	5 (10.4%)	
VA (Vancomycin)	Susceptible	45 (100%)	54 (100%)	48 (100%)	—
	Resistant	0 (0.0%)	0 (0.0%)	0 (0.0%)	
T (Tetracycline)	Susceptible	28 (62.2%)	25 (46.3%)	32 (66.7%)	0.089
	Resistant	17 (37.8%)	29 (53.7%)	16 (33.3%)	
TIG (Tigecycline)	Susceptible	45 (100%)	54 (100%)	46 (95.8%)	0.124
	Resistant	0 (0.0%)	0 (0.0%)	2 (4.2%)	
FA (Fusidic acid)	Susceptible	30 (66.7%)	23 (42.6%)	14 (29.2%)	**0.001**
	Resistant	15 (33.3%)	31 (57.4%)	34 (70.8%)	
SXT (Trimethoprim–sulfamethoxazole)	Susceptible	43 (95.6%)	51 (94.4%)	45 (93.8%)	0.928
	Resistant	2 (4.4%)	3 (5.6%)	3 (6.2%)	

Notes: *p* values were calculated using the chi-square or Fisher’s exact test, as appropriate. Bold values indicate statistical significance (*p* < 0.05). *n*: number of isolates.

## Data Availability

The datasets generated and/or analyzed during the current study are available from the corresponding author on reasonable request.

## Abbreviations

The following abbreviations are used in this manuscript: AMD: age-related macular degeneration; VEGF: vascular endothelial growth factor; Anti-VEGF: anti-vascular endothelial growth factor; EMB: eosin methylene blue; SDA: sabouraud dextrose agar; PYR: pyrrolidonyl arylamidase; AST: antimicrobial susceptibility testing; MIC: minimum inhibitory concentration; EUCAST: European Committee on Antimicrobial Susceptibility Testing; CoNS: coagulase-negative staphylococci; *S. epidermidis*: *Staphylococcus epidermidis*; *S. aureus*: *Staphylococcus aureus*; *S. hominis*: *Staphylococcus hominis*; *S. haemolyticus*: *Staphylococcus haemolyticus*; *S. lugdunensis*: *Staphylococcus lugdunensis*; *S. warneri*: *Staphylococcus warneri*; FOX: Cefoxitin; OXA: Oxacillin; LEV: Levofloxacin; E: Erythromycin; DA: Clindamycin; LNZ: Linezolid; DAP: Daptomycin; VA: Vancomycin; T: Tetracycline; TIG: Tigecycline; FA: fusidic acid; SXT: trimethoprim/sulfamethoxazole.
